# Early β adrenoceptor dependent time window for fear memory persistence in APPswe/PS1dE9 mice

**DOI:** 10.1038/s41598-020-79487-5

**Published:** 2021-01-13

**Authors:** Smitha Karunakaran

**Affiliations:** Centre for Brain Research, SID Complex, Indian Institute of Science, Bangalore, 560 012 India

**Keywords:** Neuroscience, Systems biology

## Abstract

In this study we demonstrate that 2 month old APPswe/PS1dE9 mice, a transgenic model of Alzheimer’s disease, exhibited intact short-term memory in Pavlovian hippocampal—dependent contextual fear learning task. However, their long-term memory was impaired. Intra-CA1 infusion of isoproterenol hydrochloride, the β-adrenoceptor agonist, to the ventral hippocampus of APPswe/PS1dE9 mice immediately before fear conditioning restored long-term contextual fear memory. Infusion of the β-adrenoceptor agonist + 2.5 h after fear conditioning only partially rescued the fear memory, whereas infusion at + 12 h post conditioning did not interfere with long-term memory persistence in this mouse model. Furthermore, Intra-CA1 infusion of propranolol, the β-adrenoceptor antagonist, administered immediately before conditioning to their wildtype counterpart impaired long-term fear memory, while it was ineffective when administered + 4 h and + 12 h post conditioning. Our results indicate that, long-term fear memory persistence is determined by a unique β-adrenoceptor sensitive time window between 0 and + 2.5 h upon learning acquisition, in the ventral hippocampal CA1 of APPswe/PS1dE9 mice. On the contrary, β-adrenoceptor agonist delivery to ventral hippocampal CA1 per se did not enhance innate anxiety behaviour in open field test. Thus we conclude that, activation of learning dependent early β-adrenoceptor modulation underlies and is necessary to promote long-term fear memory persistence in APPswe/PS1dE9.

## Introduction

Progressive impairment of memory and subsequent cognitive decline are a major feature of Alzheimer’s disease (AD)^1,2^. Several studies with AD patients and familial AD mouse models have suggested that memory deficits might be caused by ineffective encoding and consolidation of new information^3–6^, or disrupted retrieval of stored information^7–9^. However, how early consolidation processes influence late long-term memory persistence still remained unclear. Changes in the noradrenergic system have been observed early in the progression of AD^10–16^. Progressive damage to noradrenergic terminals and the locus coeruleus neuronal cell body have been described in multiple APP transgenic models^17–20^. In all these mouse models, degenerative changes in the locus coeruleus were observed well after the onset of plaque deposition. Interestingly, in the APPswe/PS1dE9 (APP/PS1) mice, exposure of the monoaminergic synaptic terminals or distal axons, especially to oligomeric species of Aβ^21,22^, could lead to early cortical/hippocampal pathology. APP/PS1 mice exhibit particularly widespread and severe loss of monoaminergic axons^23,24^. In this study we examined the repercussions of early damage to noradrenergic axon terminals projecting to the hippocampus in 2-month-old APP/PS1 mice. Noradrenaline released in hippocampus is known to modulate synaptic plasticity and memory consolidation through activation of β adrenergic receptors (β-AR)^25^. It has been recently demonstrated that long-term fear memory at 24 h is impaired in 2-month old APP/PS1 mice prior to onset of amyloidosis^26^. Progressive plaque deposition is known to occur at approximately 6 months of age in this mouse model^27^, and is known to parallel behavioural decline^28–30^.

Long-term persistence of fear memories is dependent on the early and late consolidation windows in the hippocampus. This process is regulated by local β-AR signalling between + 2 and + 6 h^31,32^ and D1/5 dopamine receptor signalling at the ventral hippocampus (vH) + 12 h^33–35^ post learning acquisition. The duality of function of hippocampus is attributed along its dorso-ventral axis. Noradrenergic innervation and the endogenous levels of noradrenaline are higher in vH than in dH^36,37^. The dorsal hippocampus is involved in cognitive processes and vH in emotional or anxiety related behaviour^38,39^. Distinct subsets of vCA1 neurons to basal amygdala are necessary for contextual fear learning and memory^40,41^. vCA1 also sends dense, non-overlapping projections to hypothalamus^42,43^ modulating innately anxiogenic behaviours. Therefore, distinct representation arise within the vCA1 based on the valence of the stimuli (innate vs learned) routing information via projection defined vCA1 neurons^40^. However, we still do not know the dynamics and the nature of learned fear versus innate anxiety in APPswe/PS1dE9 mice at an age where there is constitutive overexpression of APP and PS1 genes without β-amyloid deposition.

Here we applied contextual fear conditioning (cFC) and open field test (OFT) to investigate the causal role of local vH β-AR dependent signalling in modulating learned fear versus innate anxiety related behaviour. The current study primarily mapped the time course of fear expression after cFC in APP/PS1 mice. Consequently, this measure has proved useful in detecting the time dependent decline in the strength of fear memory after learning acquisition. Further experiments were designed specifically to study the role of β-AR dependent plasticity in the vH during early stages of memory consolidation, that would determine long-term fear memory persistence in 2-month-old APP/PS1 mice. We report that APP/PS1 has a local vH β-AR signalling dependent time window spanning from 0 to + 2.5 h, which is imperative for long-term fear memory persistence. Furthermore, using OFT, we demonstrate that vH β-AR agonist infusion per se does not interfere with innate anxiety responses of APP/PS1 mice.

## Materials and methods

### Experimental animals

The generation, care, and use of mice as well as all experimental procedures were approved by the Institutional Animal Ethics Committee of the Indian Institute of Science, Bangalore. Transgenic mice B6C3-Tg (APPswe/PS1dE9) 85Dbo/J (https://www.jax.org/strain/005864) were originally obtained from The Jackson Laboratory, and was kindly provided by Prof. Vijayalakshmi Ravindranath, Director, Centre for Brain Research, Bangalore, India^26^. Wild Type (WT) and APPswe/PS1dE9 (APP/PS1) mice were bred at the Institutional Central Animal Facility, and were housed in standard mouse cages under conventional laboratory conditions (12 h dark and 12 h light cycle, constant temperature and humidity), and were given food and water ad libitum. Behavioural experiments were performed using male APP/PS1 and WT mice. No samples were excluded from any of the experiments described herein, unless otherwise mentioned in the analysis. Animal experiments were designed and followed in compliance with the Animal Research: Reporting of In Vivo Experiments (ARRIVE) guidelines. WT and APP/PS1 mice were assigned randomly to the respective groups based on the genotype. Different litters of the same age group were taken and were divided into control and experimental groups.

### Behavioural procedures

All experiments were carried out with male mice that were 55–65 days old (~ 30 g) at the beginning of the experiment. Mice were housed individually for 3 days, and were handled for 5 min everyday prior to behavioural testing. All behavioural experiments were conducted at approximately the same time during the light cycle (9:00–15:00) by one constant experimenter.

### Contextual fear conditioning (cFC)

For cFC^26^, the training context was rectangular in shape. Identity of the context was maintained with the presence of 2% acetic acid (vol/vol). The conditioning chamber was cleaned with 70% ethanol before and after each session. Mice were allowed to explore the apparatus for 1 min, and then received 3 foot shocks (2 s and 0.6 mA each, intershock interval 30 s). Contextual fear memory was assessed by returning the mice to the training context 24 h or one month after fear conditioning and analysing freezing during a test period of 2 min.

### Open field test (OFT)

Mice were released in the corner of a 45 × 45 cm open field arena (Fig. 3a). Their activity was recorded with a camera mounted on the ceiling above the center of the open field arena for 10 min. At the end of testing, mice were returned to their home cage. The arena was partitioned into outer periphery and center region, and the amount of time spent and the distance travelled were extracted using a previously validated open-source toolbox for automated phenotyping of mouse behaviour^44^.

### Pharmacology in vivo

All the local treatments were carried out with the help of cannulas (33G). Guide cannulae (26 gauge, Plastics One) were implanted to the skull with dental cement. Mice were then given 1 week to recover from surgery. 32-gauge stainless steel injectors attached to 5-μl Hamilton syringes were inserted into the guide cannulae to deliver the following drugs—Isoproterenol Hydrochloride (0.25 μg per side; Sigma Aldrich, India); ( ±)-Propranolol hydrochloride (0.5 μg per side; Sigma Aldrich, India), and 0.9% saline. Coordinates relative to bregma are as follows: vH (AP − 3.0, ML ± 2.6, DV − 3.2). A total volume of 200 nl was injected; the injector was left for another minute to allow diffusion into the tissue.

### Statistical analysis

Data analysis was performed using Prism 7 (GraphPad Software Inc., La Jolla, CA, USA). No statistical methods were used to predetermine sample sizes. Our sample sizes are similar to those generally employed in the field^33^. The sample size per group is mentioned in the respective figure legends. Statistical analyses were designed using the assumption of normal distribution and similar variance among groups. They were performed using unpaired two-tailed Student’s t-test for paired comparisons, and two-way ANOVA followed by post hoc tests was performed when two factors were compared. Results are presented as mean ± S.E.M. The significance of the results was accepted at *P* < 0.05. Eta squared (η2) are reported as a measure of effect size^45^.

## Results

### 2-month-old APP/PS1 mice exhibit long-term, but not short-term fear memory loss

**Figure 1:**
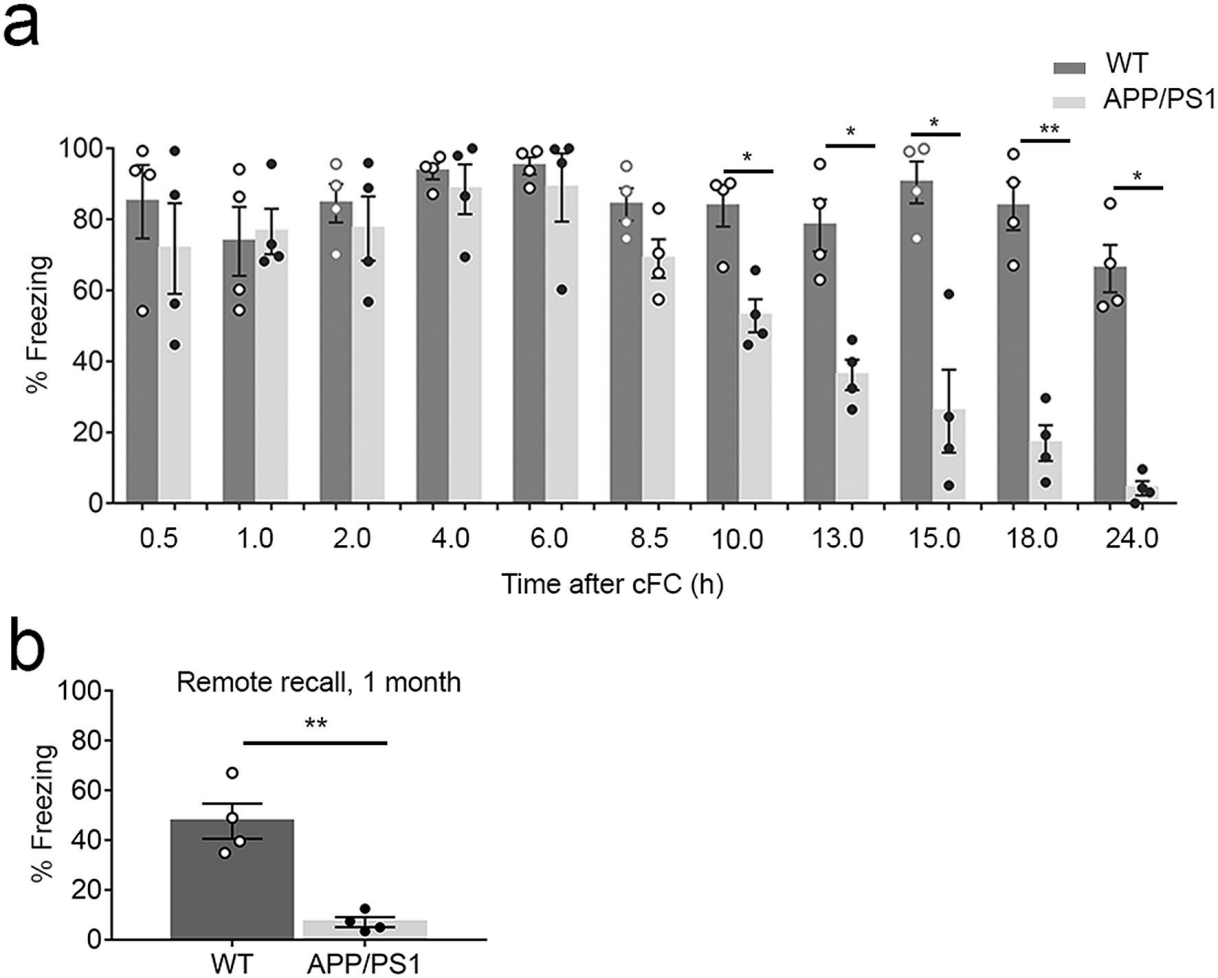
Long-term fear memory, not short-term is impaired in 2-month-old APP/PS1 mice: (**a**) Time course of percentage time spent freezing to the context during memory recall at different time points post fear conditioning. There is a gradual decline of strength of fear memory + 10 h onwards in APP/PS1 mice compared to WT. n = 4, Two-way ANOVA followed by Sidak’s post hoc test. (**b**) Percentage time spent freezing to the context during remote memory recall after one month of cFC. n = 4; Two tailed unpaired Students t test; Data are presented as mean ± S.E.M. *P = 0.0332, **P = 0.0021.

In order to determine whether freezing impairments observed at 24 h in 2-month-old APP/PS1^26^ are due to a failure to form short-term memory, we tested APP/PS1 for recall of fear at different time points post cFC from + 0.5, + 1, + 2, + 4, + 6, + 8.5, + 10, + 13, + 15, + 18 till + 24 h. (+) indicates the timepoint after cFC. This experiment was conducted with 11 groups of WT and APP/PS1 mice, with n = 4 mice per group per time point. We designated the time at which the learning protocol was completed (in cFC: the last of the three foot shocks in the training context) as the time of acquisition, or time + 0 h. Freezing was intact in APP/PS1 mice tested between + 0.5 and + 8.5 h, indicating an intact short-term memory. However, strength of the memory displayed a steep decline from + 10 h till + 24 h in APP/PS1 mice as compared to WT (Fig. 1a: Two-way ANOVA followed by Sidak’s post hoc test: time effect: F (3.466, 20.80) = 12.66, *P* < 0.0001; genotype effect: F (1, 6) = 108.2, *P* < 0.0001; interaction: F (10, 60) = 7.287, *P* < 0.0001). Recall at a remote time point (1 month) post cFC acquisition also indicated a weak fear memory in APP/PS1 as compared to WT mice (Fig. 1b: two-tailed unpaired Student's t test; 1 month : t_(6)_ = 5.503,* P* = 0.0015, η2 = 0.8346). These results clearly indicate that short-term memory is intact in 2-month-old APP/PS1 mice.

### β-AR activation during acquisition or immediately post acquisition promotes long-term fear memory persistence in APP/PS1 mice

**Figure 2:**
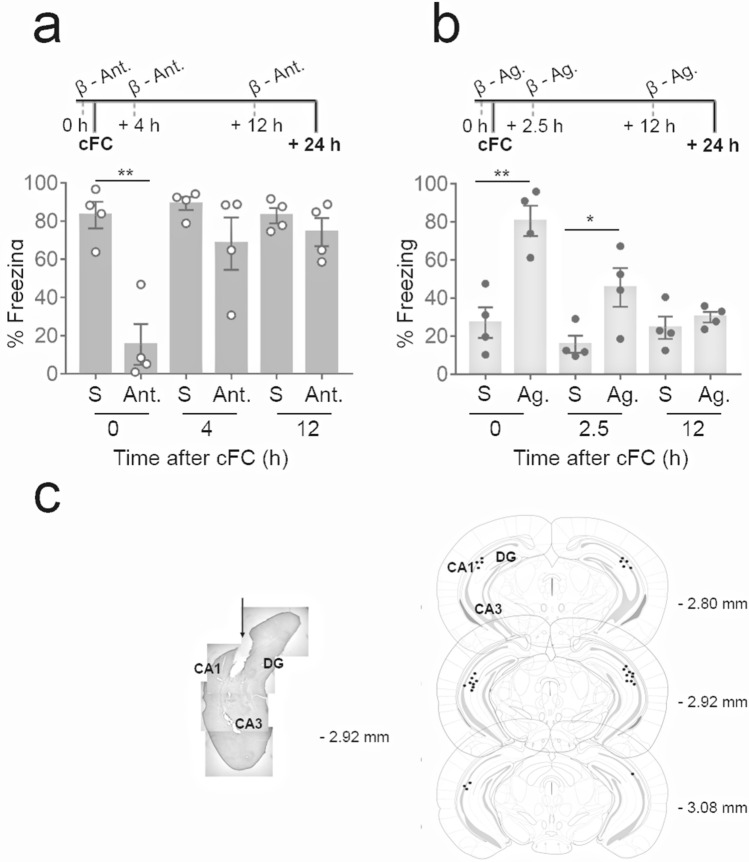
Infusion of β-adrenergic receptor agonist at—0 h modulates strength of memory in 2-month-old APP/PS1 mice: (**a**) β-AR antagonist delivery to vH at + 0 h, but not at + 4 h or + 12 h decreased freezing in the WT mice (S: 0.9% Saline; Ant: β-AR antagonist). (**b**) Time windows at which local delivery of β-AR agonist to vH influence long-term consolidation of fear memory (S: 0.9% Saline; Ag: β-AR agonist) in APP/PS1 mice. Long-term fear memory consolidation specifically depends on vH β-AR signaling around + 0 h. Infusion of β-AR agonist at + 2.5 h, but not at + 12 h post cFC partially rescues fear memory in APP/PS1 mice. (**c**) Mouse brain coronal section (left) with location of vH (DG, CA3, CA1). To the right, numbers: antero-posterior coordinates caudal to bregma. Representation of the tips of the injectors (black circles) aiming the vH CA1. Injection sites from 15 mice into CA1 region of vH. n = 4; Two tailed unpaired Students t test; Data are presented as mean ± S.E.M. *P = 0.0332, **P = 0.0021.

To determine whether endogenous local β-AR signalling is necessary to ensure longevity of short-term memory during and following learning, we delivered β-AR antagonist (±)-Propranolol hydrochloride to the vH CA1 (Fig. 2c) at different time points in APP/PS1 mice (Fig. 2a). A previous report suggested that the injection of protein synthesis inhibitor anisomycin to the CA1 impaired long-term memory supporting the existence of an early phase of synaptic plasticity occurring at mossy fiber terminals during cFC^46^. Moreover, the vH was chosen due to its reciprocal connections to the amygdala, mediating fear memory^38–41,46,47^, and due to its early involvement in fear memory consolidation and expression unlike dorsal hippocampus^33,48^. β-AR antagonist delivered to vH CA1 10 min before acquisition (− 10 min) prevented long-term memory consolidation in WT mice (Fig. 2a: two-tailed unpaired Student’s t test; + 0 h: t_(6)_ = 5.331, *P* = 0.0018, η2 = 0.8257). However, β-AR antagonist delivery did not suppress long-term fear memory when delivered at + 4 h and + 12 h after acquisition (Fig. 2a: two-tailed unpaired Student's t test; + 4 h: t_(6)_ = 1.484, *P* = 0.1884, η2 = 0.2684; + 12 h: t_(6)_ = 1.026, *P* = 0.3443, η2 = 0.1494). Furthermore, β-AR agonist delivered to vH immediately before acquisition at + 0 h produced increased freezing to context at + 24 h compared to saline treated APP/PS1 animals (Fig. 2b: two-tailed unpaired Student's t test; + 0 h: t_(6)_ = 4.714, *P* = 0.0033, η2 = 0.7874), and were undistinguishable from fear memory of WT mice. However, β-AR agonist delivery at + 2.5 h only partially enhanced the persistence of long-term fear memory (Fig. 2b: two-tailed unpaired Student’s t test; + 2.5 h: t_(6)_ = 2.684, *P* = 0.0364, η2 = 0.5455; + 0 h vs. + 2.5 h: t_(6)_ = 2.693, *P* = 0.0359, η2 = 0.5473). Furthermore, the β-AR agonist failed to rescue long-term fear memory persistence when delivered + 12 h after acquisition (Fig. 2b: two-tailed unpaired Student’s t test; + 12 h: t_(6)_ = 0.8798, *P* = 0.4128, η2 = 0.1143). Previous studies have shown that, under local in vivo delivery conditions comparable to those used here, the agonist is effective up to 2.5–5 min following delivery^49^. These results clearly indicate that long-term memory persistence is determined by a unique early time window (+ 0 to + 2.5 h) in the vH CA1 post learning acquisition in APP/PS1 mice. Moreover, it indirectly indicates early noradrenergic dysfunction in this animal model.

### β-AR activation did not interfere with innate anxiety-related behaviour in APP/PS1 mice

**Figure 3:**
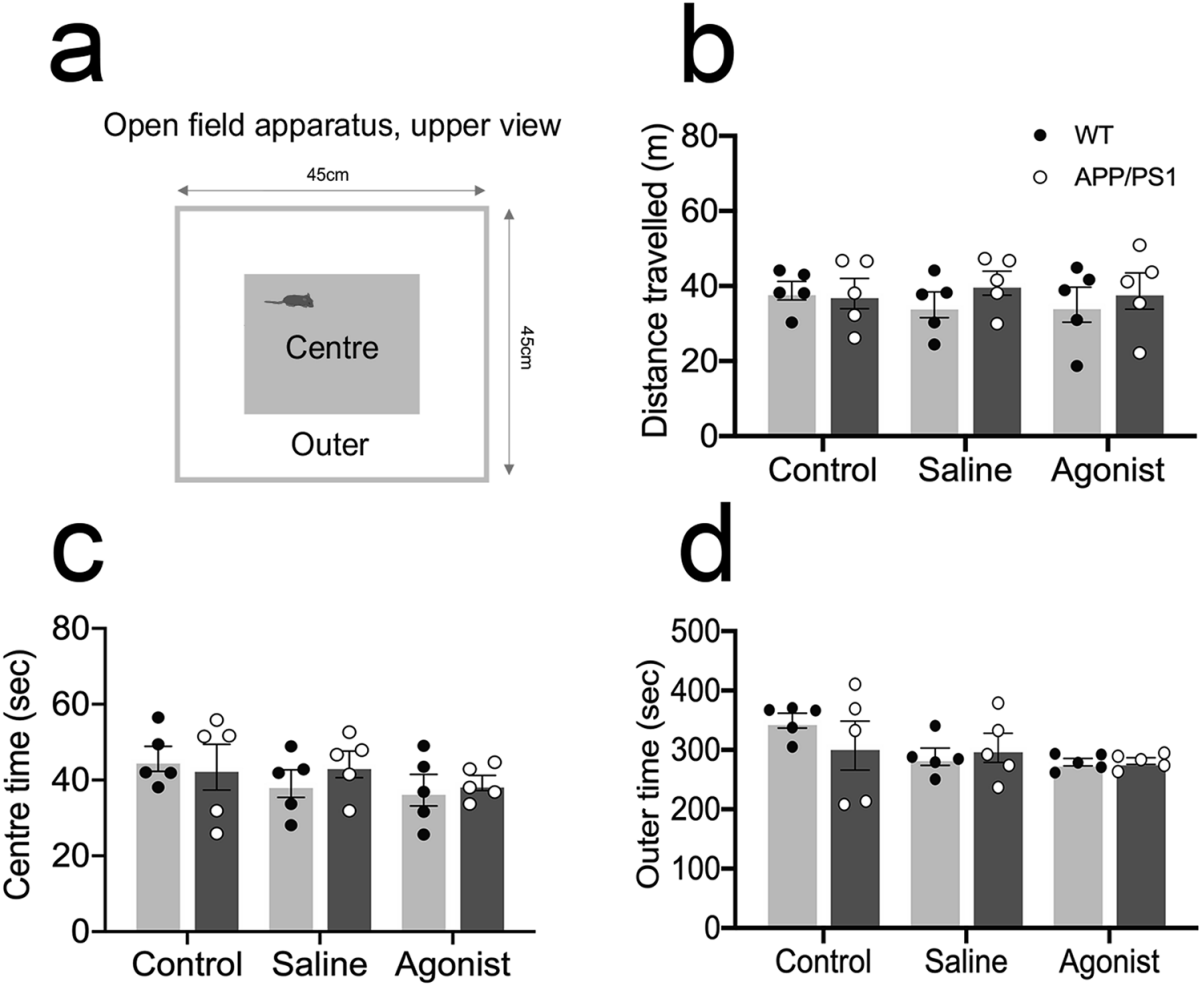
Infusion of β-adrenergic receptor agonist at—0 h do not modulate innate anxiety levels in 2-month-old APP/PS1 mice: (**a**) Open-field test apparatus. WT and APP/PS1 mice were tested for distance travelled (**b**), time spent in the center (**c**), and time spent in the outer periphery (**d**). n = 5; two-way ANOVA followed by Sidak’s post hoc test. Data are presented as mean ± S.E.M.

The OFT assess anxiety-like behaviours in response to a novel environment. Distance travelled is a measure of ambulatory movement, whereas the amount of time spent in the center zone versus the outer zone is a measure of anxiety levels due to the rodent’s natural aversion or avoidance behaviour to open spaces^50^. Two-way ANOVA followed by Sidak’s post hoc test did not reveal any significant differences between APP/PS1 and WT mice, in the amount of time each mouse spent in the open center zone (Fig. 3c: treatment effect: F (2, 24) = 1.232, *P* = 0.3096; genotype effect: F (1, 24) = 0.2396, *P* = 0.6289; interaction: F (2, 24) = 0.4229, *P* = 0.6599), the outer zone (Fig. 3d: treatment effect: F (2, 24) = 2.632, *P* = 0.09626; genotype effect: F (1, 24) = 0.2594, *P* = 0.6152; interaction: F (2, 24) = 0.9806, *P* = 0.3896) and the total ambulatory movement (Fig. 3b: treatment effect: F (2, 24) = 0.08025, *P* = 0.9231; genotype effect: F (1, 24) = 0.8361, *P* = 0.3696; interaction: F (2, 24) = 0.3744, *P* = 0.6916) following β-AR agonist delivery to vCA1 immediately before taking the mice to OFT.

## Discussion

In this study, we describe the temporal course of behavioural expression of fear memory upon cFC, to determine the dynamics of β-AR modulation and memory consolidation in the vH CA1 of 2-month old APP/PS1 mice. We demonstrate a unique β-AR dependent time window of early memory consolidation (+ 0–2.5 h) that is crucial for long-term fear memory persistence in APP/PS1 mice. Our findings are consistent with previous reports using 7-month-old APP/PS1 mice that are impaired in long-term but not in short-term contextual fear memory^9^. The APP/PS1 mouse model exhibits constitutive overexpression of APP and PS1 genes, elevated levels of toxic soluble oligomeric Aβ, and synaptic deficits are reported at 1–1.5^51^ and 3.5 months of age in the cortex and hippocampus^52–54^. This might underlie the mild behavioural impairments observed in our study with 2-month-old APP/PS1 mice. We further demonstrate that β-AR agonist per se does not modulate the baseline innate anxiety levels of APP/PS1.

Pharmacological studies using male Sprague–Dawley rats have indicated that between 0 and 6 h after a learning experience, the noradrenergic system is activated to reinforce long-term memory processing in the hippocampus^31,32^ and prefrontal cortex^55–57^. Our results indicate the existence of a local β-AR signalling dependent early consolidation window between + 0 and 2.5 h which is required to ensure long-term fear memory persistence. Consistent with this notion, the late consolidation window which has been reported to be D1/5R dependent at + 12 h^33–35^ was unaffected by administration of β-AR antagonist.

Apart from being involved in consolidation of long-term fear memory, vH neurons also carry a representation of innately anxiogenic stimuli. Manipulation of the vH afferents or efferents are also known to directly impact anxiety-related behaviour^58–60^. Furthermore, anxiolytic effects of propranolol have been well documented in animal models of anxiety^61,62^. Therefore, the increased freezing to context induced by β-AR agonist may have emerged from β-AR agonist induced changes in innate anxiety levels of APP/PS1. This led us to perform open field experiment with APP/PS1 mice following vCA1 β-AR agonist infusion to investigate whether the β-AR agonist potentiated the consolidation of the fear memory trace, or enhanced innate anxiety levels to promote avoidance behaviour. Intriguingly, we found the concentration of β-AR agonist which we administered in our studies did not have a significant role in mediating an avoidance behaviour to the context in APP/PS1.

It was recently demonstrated that β2-AR expressed in astrocytes are the primary mediators of fear based contextual memory consolidation, rather than β1-AR which are primarily located in the neurons^63^. These findings indicate the differential contribution of β1-ARs and β2-ARs in mediating hippocampal memory formation and associated processes. More importantly, β2-AR dysregulation in astrocytes has been implicated in AD^64^. These findings further suggest that the β-AR agonist may facilitate strengthening of memory trace by gating synaptic plasticity in vCA1 synapses through distinct pathways for learned versus innate anxiety, based on the affective valence of the inputs.

Memory stabilization after learning also involves temporal molecular changes^65^. De novo protein synthesis occurring within 1 h or around 4 h after learning acquisition is known to be important for memory stabilization^66^. A recent study^67^ demonstrated translational repression of specific genes such as neurensin-1 (Nrsn1) and Mitogen-activated protein kinase 6 (Mapk6) around 5–10 min post cFC acquisition without a change in mRNA levels. Furthermore, at later time points of 30 min and 4 h, they observed gene suppression especially of a group of genes which were positively controlled by Estrogen Receptor 1 (ESR1). Overexpressing Nrsn1 or activating ESR1 in the hippocampus post learning acquisition was sufficient to impair contextual fear memory formation. Interestingly, signalling pathways of ESRs are intertwined with those of the β-ARs^68^ pointing to the possibility of functional convergence and their interdependence at around 2.5 h in the vH modulating long-term memory consolidation. However, we still do not know the mechanisms underlying these upstream regulations which might be pertinent for long-term memory persistence.

The most progressive hypothesis with regard to memory deficits in AD is the ‘retrieval deficit’ where the store of memory remains relatively intact during early AD and the deficits are associated with inability to access and modify this information long-term^7–9^. In contrast, our results support the notion that early consolidation deficits caused by absence of appropriate β-adrenergic neuromodulation may be responsible for memory deficits in early AD. Thus, β-adrenergic neuromodulation may be necessary to recruit and maintain system wide networks for long-term memory persistence in APP/PS1 mice. Accordingly, the unique time window for memory persistence uncovered in this study may represent a new therapeutic window to alter memory networks and promote better quality control of mechanisms that are required for long-term memory consolidation.

## Data Availability

Requests for raw data can be addressed to the corresponding author.
